# In health research publications, the number of authors is strongly associated with collective self-citations but less so with citations by others

**DOI:** 10.1186/s12874-023-02037-w

**Published:** 2023-10-11

**Authors:** Cyril Jaksic, Angèle Gayet-Ageron, Thomas Perneger

**Affiliations:** 1 UAM-CRC & Division of Clinical Epidemiology, Department of health and community medicine, University of Geneva & University Hospitals of Geneva, Geneva, Switzerland; 2 Present Address: Hôpitaux Universitaires de Genève, Service d’épidémiologie Clinique, Bvd de la Tour 8, Geneva, 1211 Switzerland; 3 Division of Clinical Epidemiology, Department of health and community medicine, University of Geneva & University Hospitals of Geneva, Geneva, Switzerland

**Keywords:** Citation analysis, Self-citations, Research quality metrics, Indicators

## Abstract

**Objective:**

This study investigated the associations between the number of authors and collective self-citations versus citations by others.

**Study design and setting:**

We analyzed 88,594 health science articles published in 2015 and citations they received until 2020. The main variables were the number of authors, the number of citations by co-authors (collective self-citations), and the number of citations by others.

**Results:**

The number of authors correlated more strongly with the number of citations by co-authors than with citations by others (Spearman r 0.31 vs. 0.23; mutually adjusted r 0.26 vs. 0.12). The percentage of self-citations among all citations was 10.6% for single-authored articles, and increased gradually with the number of authors to 34.8% for ≥ 50 authors. Collective self-citations increased the proportion of articles reaching or exceeding 30 total citations by 0.7% for single-authored articles, but by 11.6% for articles written by ≥ 50 authors.

**Conclusions:**

If citations by others reflect scientific utility, then another mechanism must explain the excess of collective self-citations observed for multi-authored articles. The results support the hypothesis that the authors’ own motivations explain this excess. The evaluation of scientific utility should also be based on citations by others, excluding collective self-citations.

**Supplementary Information:**

The online version contains supplementary material available at 10.1186/s12874-023-02037-w.

**Table:** 

**What is new?**
• The number of co-authors was strongly associated to collective self-citations.
• The number of co-authors was weakly related to citations by others.
• Collective self-citations represented more than 30% of total citations for multi-authored (≥ 30) articles.
• An h-index calculated without collective self-citations should be reported along with the traditional h-index.

## Introduction

In the scientific literature, citations are used to support scientific statements, to reference theories and methods, and to put research results in perspective [1–3]. Articles that are particularly useful to others are cited frequently, less useful articles less so. For this reason, the number of citations of a particular article is commonly treated as an indicator of its quality, or at the least, of scientific utility [1–3]. As a result, it is thought that a researcher’s scientific contribution can be assessed, for promotion for instance, by metrics relying on the number of citations, like the h-index.

Because researchers typically explore the same scientific theme in consecutive studies, they regularly cite their own previously published papers [3–5]. Such self-citations may simply ensure continuity among the publications and avoid duplication, regardless of utility to the wider scientific community. This self-referential usage of citations introduces noise into the interpretation of citation counts (and of derived metrics such as the h-index) as indicators of research utility, particularly when self-citations represent a large proportion of total citations. An additional concern is that self-citations are vulnerable to manipulation [4, 5]. E.g., many Italian researchers have increased their rate of individual self-citation following the introduction of a national research assessment scheme in 2011-12 that relied on citations [6]. It is useful to distinguish between individual self-citations (i.e., citations to a particular paper made by one of its co-authors) and collective self-citations (i.e., the sum of citations made by all co-authors).

A natural property of collective self-citations that is unrelated to scientific utility is that they automatically increase with the number of co-authors [4, 7–9]. Each of the co-authors may have good reason to cite the published study a certain number of times, and the more authors there are, the more collective self-citations will accrue. Nevertheless, previous studies have concluded that the number of authors is only weakly associated with the number of self-citations [4, 8], and that this does not meaningfully distort research assessment. However, these studies date from an era when very long author lists were uncommon; indeed, both studies analyzed papers with only up to 15 co-authors. Currently, research papers are more commonly co-signed by larger number of authors [9].

A positive association between the number of authors and collective self-citations would not in itself imply bias in the assessment of scientific value. Multi-authored papers might provide greater diversity and have higher impact than papers written by fewer authors, and thus receive more citations – including collective self-citations – due to their greater scientific utility [10, 11]. Indeed, oft-cited articles reporting on large clinical trials, multi-national collaborations or genome-wide association studies, typically have numerous co-authors. A positive association between the number of authors and the total number of citations has been described previously [12–19].

**Fig. 1:**
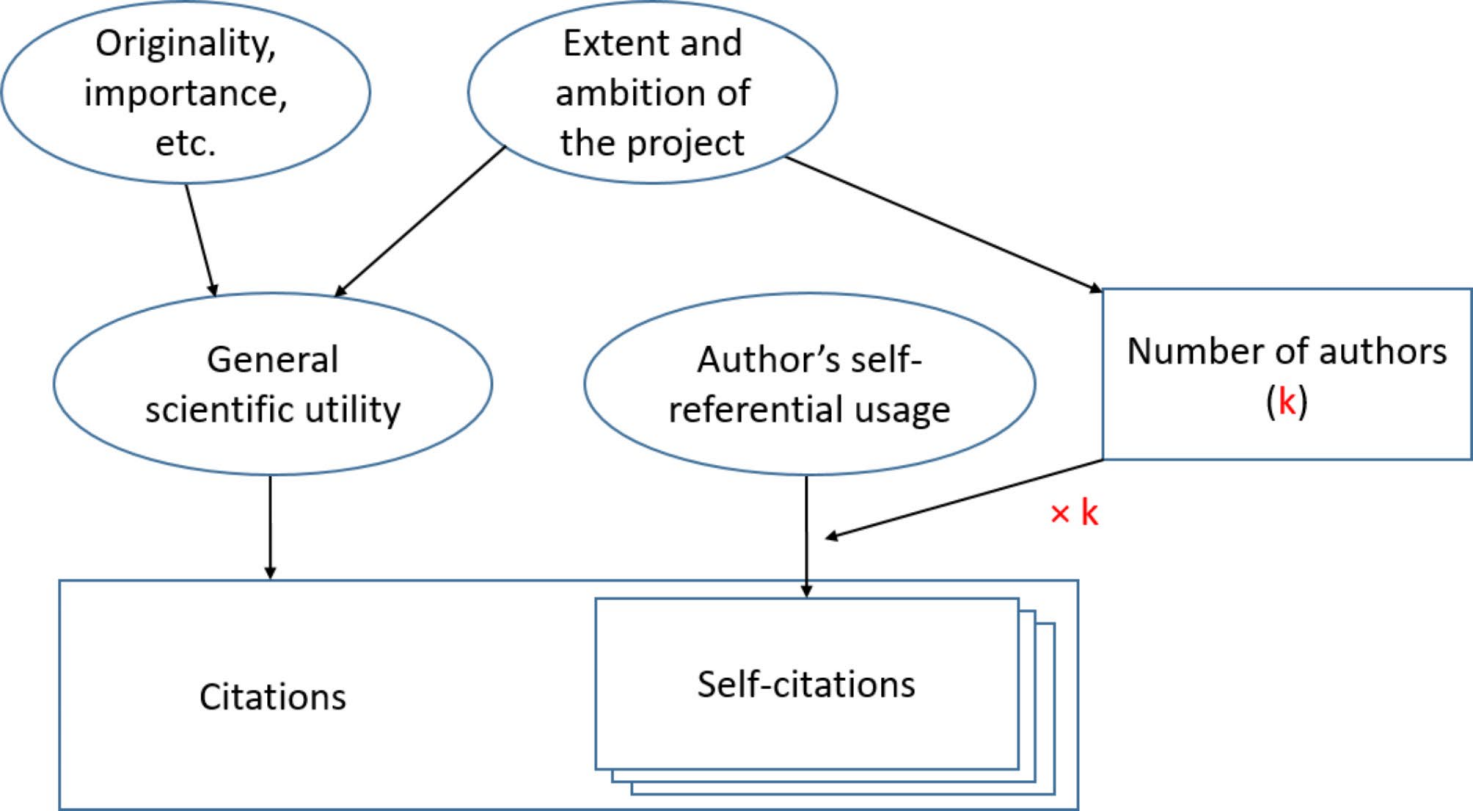
Conceptual model: an ambitious study is more likely to have greater general scientific utility, and may require more extensive scientific collaboration, hence a greater number of co-authors. General scientific utility drives all citations – citations by others, but also self-citations. Authors’ self-referential usage drives self-citations only. Rectangles represent observed variables; ovals represent latent variables. Collective self-citations encompass the self-citations by all *k* co-authors

In this study, we sought to clarify the relationships between the number of authors and the number of citations, comparing collective self-citations to citations by others, among papers published in the health sciences. We hypothesized that the number of authors would be associated with citation counts through two possible mechanisms (Fig. 1). First, multi-authored articles may have ambitious scientific objectives and achieve greater scientific utility, which would increase the number of both citations by others and collective self-citations. Second, multi-authored articles may have a greater potential for collective self-citations, because a larger pool of authors may need to reference the study in ensuing work.

## Methods

We conducted a prospective study of original articles in the health sciences published in 2015 and indexed in Scopus, and their subsequent citations in years 2015 to 2020. We chose 2015 to 2020 to have sufficient lag for citations.

The included papers were original research articles (tagged as “articles” in Scopus) appearing in journal issues published between January the 1st and December 31st 2015, in health-related fields, i.e., in journals with selected All Science Journal Classification (ASJC) codes [20] (see Appendix 1). The ASJC codes retained for the present study were those associated with fields pertaining to medical and clinical applications (excluding fields related to basic sciences or non-medical specialties). In addition, to focus on journals reflecting a certain impact in sciences, we used the *CiteScore* metrics [21] provided by Scopus, mirroring that of the impact factor, and selected only journals with scores above five. For each article, the counts of total citations, collective self-citations and citation by others were extracted. Collective self-citations were identified using the unique identification number assigned to each author within the Scopus system. All data were automatically extracted by the International Center for the Study of Research (ICSR) lab working in collaboration with Elsevier, owner of Scopus. The database provided by Scopus included 92,835 articles. Articles with the keywords “meta-analysis” or “systematic review” in their title were excluded (n = 1,673), in order to limit the analysis to original research articles. Due to errors or incomplete data, the sum of collective self-citations and citations by others did not always account for all citations reported by the total citation count. As a result, after initial database cleaning, we excluded 2,568 articles for which the difference between total citations and the sum of collective self-citations and citations by others exceeded 5% of the total citation count (or 2 citations if total citations were < 40), as this raised concerns about the accuracy of the data. Thus remained 88,594 articles. In this analysis, we used the number of collective self-citations, the number of citations by others, and their sum.

The main independent variable was the number of authors, split into 7 categories (1, 2–4, 5–9, 10–19, 20–29, 30–49, 50 authors and more). We obtained mean and median numbers of citations (sum of all citations, collective self-citations, citations by others) for each category of number of authors, as well as the mean and median proportion of collective self-citations. For all quantitative variables, we provided descriptive statistics by mean, standard deviation (SD), quartiles, and range. We obtained Spearman correlation coefficients between the number of authors and the two types of citations. We also computed partial Spearman correlation coefficients [22], i.e. correlations between the number of authors and each type of citation, adjusted for the other type of citation.

To quantify the potential impact of collective self-citations on authors’ h-indices, we obtained the proportions of articles which either met or exceeded the thresholds of 10, 20, 30 and 50 citations (arbitrary thresholds for the h-index), using all types of citations, across the 7 categories of author numbers; we also computed the increase in each proportion attributable to collective self-citations. Finally, we explored graphically the association between number of authors and collective self-citations, stratifying on citations by others (in strata of 0–4, 5–9, 10–14, 15–19, 20–29, 30–49, and ≥ 50 citations by others), using non-parametric regression [18]. We performed the same analysis for citations by others, stratifying on collective self-citations (in strata of 0, 1, 2–3, 4–6, 7–9, 10–14, and ≥ 15 collective self-citations). The stratifications were adapted to the distributions of each variable, post-hoc. Of note, the non-parametric regression lines show mean values of the dependent variable (citations) at a given level of the independent variable (number of authors), smoothed via weighted linear regression. We used logarithm-transformed values of citations to improve interpretation of the figures. Analyses were conducted using SPSS (v.25) and R (v.4.1.0), and graphs were done using STATA (v.17.0).

## Results

The final database contained data for 88,594 articles from 997 journals. The mean number of authors per article was 7.9 (SD 8.7, quartiles 5, 7 and 10, range 1 to 862). The mean number of citations per article was 25.5 (SD 82.5, quartiles 8, 15 and 28, range 0 to 15,846), of which the mean number of collective self-citations was 4.1 (SD 6.9, quartiles 1, 2 and 5, range 0 to 410), and the mean number of citations by others 21.4 (SD 79.7, quartiles 6, 12 and 23, range 0 to 15,786). Among 85,623 articles that received at least one citation (96.6%), the mean proportion of collective self-citations among all citations was 19.2% (SD 19.4, quartiles 3.8%, 14.3%, 28.6%, range 0 to 100%). The Spearman correlation coefficient between collective self-citations and citations by others was 0.42.

### Associations between number of authors and citation counts

The Spearman correlation coefficient between the number of authors and the number of citations by others was 0.23; and between the number of authors and number of collective self-citations 0.31. Corresponding mutually adjusted coefficients were 0.12 and 0.26 respectively. Due to the large number of observations, the 95% confidence intervals on the correlations coefficients were less than 0.02 in width.

Both the number of citations by others and the number of collective self-citations increased gradually as the number of authors increased (Table 1); only the highest category (≥ 50 authors) gathered slightly fewer citations by others than the preceding category (30–49 authors). The proportion of collective self-citations increased gradually from one ninth for single author papers to one third for papers with ≥ 50 authors (Table 1).

**Table 1:** Distribution of research articles across 7 categories of numbers of authors, and means (SD) and medians (Q1-Q3) of citations obtained in 2015-20 (Total citations, citations by others, collective self-citations, self-citations as percentage of total), and percentage of uncited papers, for 88,594 articles in health-related fields published in 2015

Number of authors	N (%)	Total citations, mean (SD) median (Q1-Q3)	Citations by others, mean (SD) median (Q1-Q3)	Collective self-citations, mean (SD) median (Q1-Q3)	Percent collective self-citations, mean (SD) median (Q1-Q3)	Uncited articles (percent)
1	2618 (3.0)	10.5 (29.8) 2 (0–11)	9.6 (28.8) 2 (0–11)	0.8 (2.5) 0 (0–11)	10.6 (20.1) 0 (0–11)	40.6
2–4	19306 (21.8)	19.9 (76.5) 12 (6–23)	17.3 (75.9) 10 (4–23)	2.6 (3.8) 1 (0–23)	16.5 (19.7) 10 (0–23)	6.2
5–9	43159 (48.7)	22.5 (81.6) 15 (8–26)	18.9 (80.6) 12 (6–26)	3.6 (4.8) 2 (1–26)	19.3 (19.4) 14 (4–26)	1.4
10–19	20956 (23.7)	32.4 (54.6) 20 (11–36)	26.7 (50.2) 15 (8–36)	5.7 (7.7) 4 (1–36)	21.4 (18.5) 17 (8–36)	0.5
20–29	1874 (2.1)	67.2 (157.9) 33 (18–69)	55.5 (145.4) 26 (13–69)	11.6 (16.7) 7 (3–69)	23.2 (17.6) 20 (11–69)	0.1
30–49	491 (0.6)	109.4 (347.0) 39 (20–87)	91.0 (325.8) 29 (14–87)	18.4 (31.3) 9 (4–87)	26.0 (18.0) 23 (12–87)	0.6
≥ 50	190 (0.2)	93.7 (339.4) 36 (15–71)	72.5 (305.4) 26 (9–71)	21.2 (39.4) 10 (4–71)	34.8 (22.8) 32 (19–71)	0.5

The proportion of uncited articles exceeded 40% for single-author articles, dropped to 6.2% for articles with 2–4 authors, 1.4% for articles with 5–9 authors, and below 1% for articles with 10 authors or more (Table 1).

**Fig. 2:**
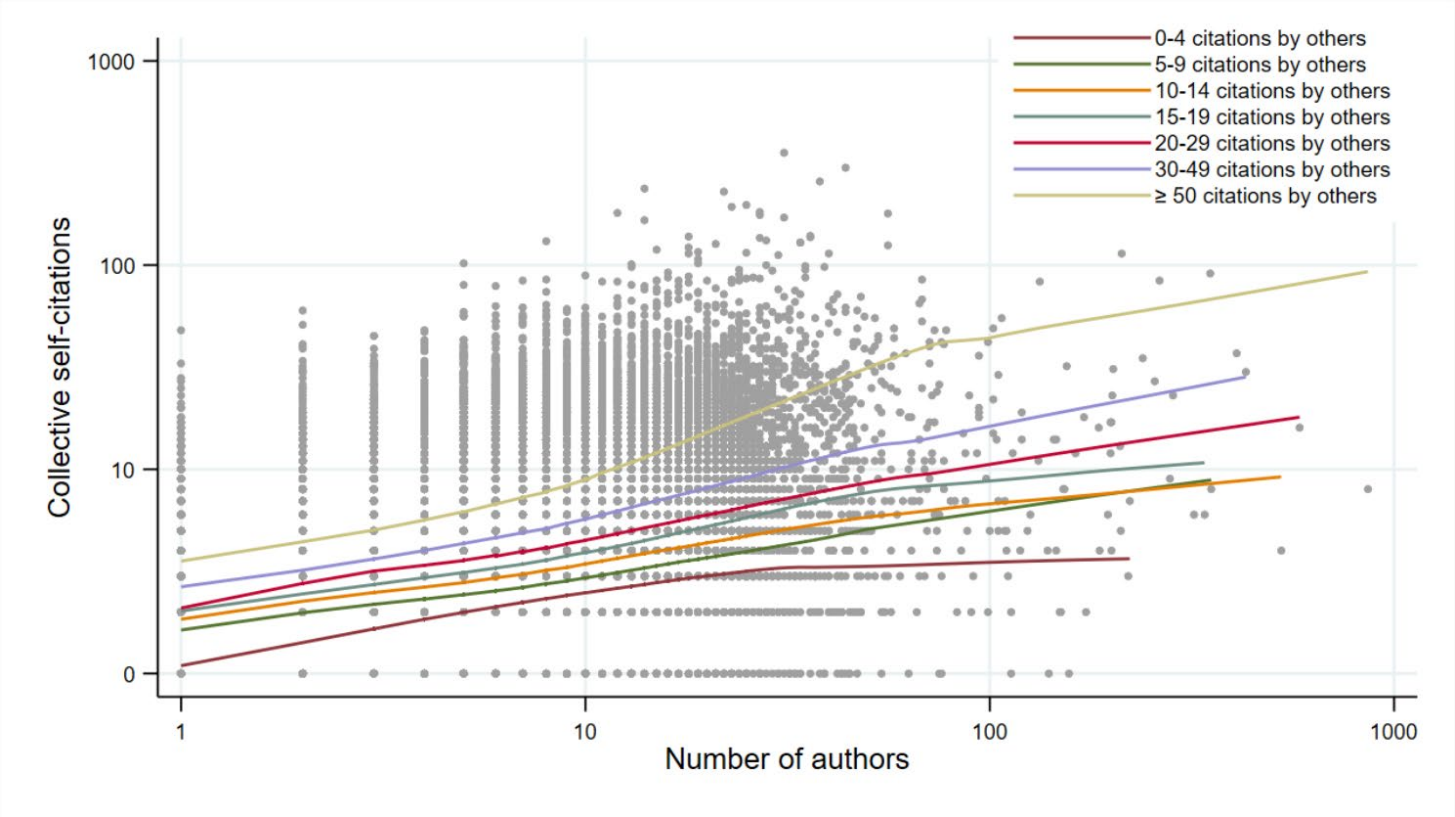
Scatter-plot of the number of collective self-citations (logarithm scale) as a function of the number of authors (logarithm scale), stratified by the number of citations by others. Lines are non-parametric regression functions (Lowess)

**Fig. 3:**
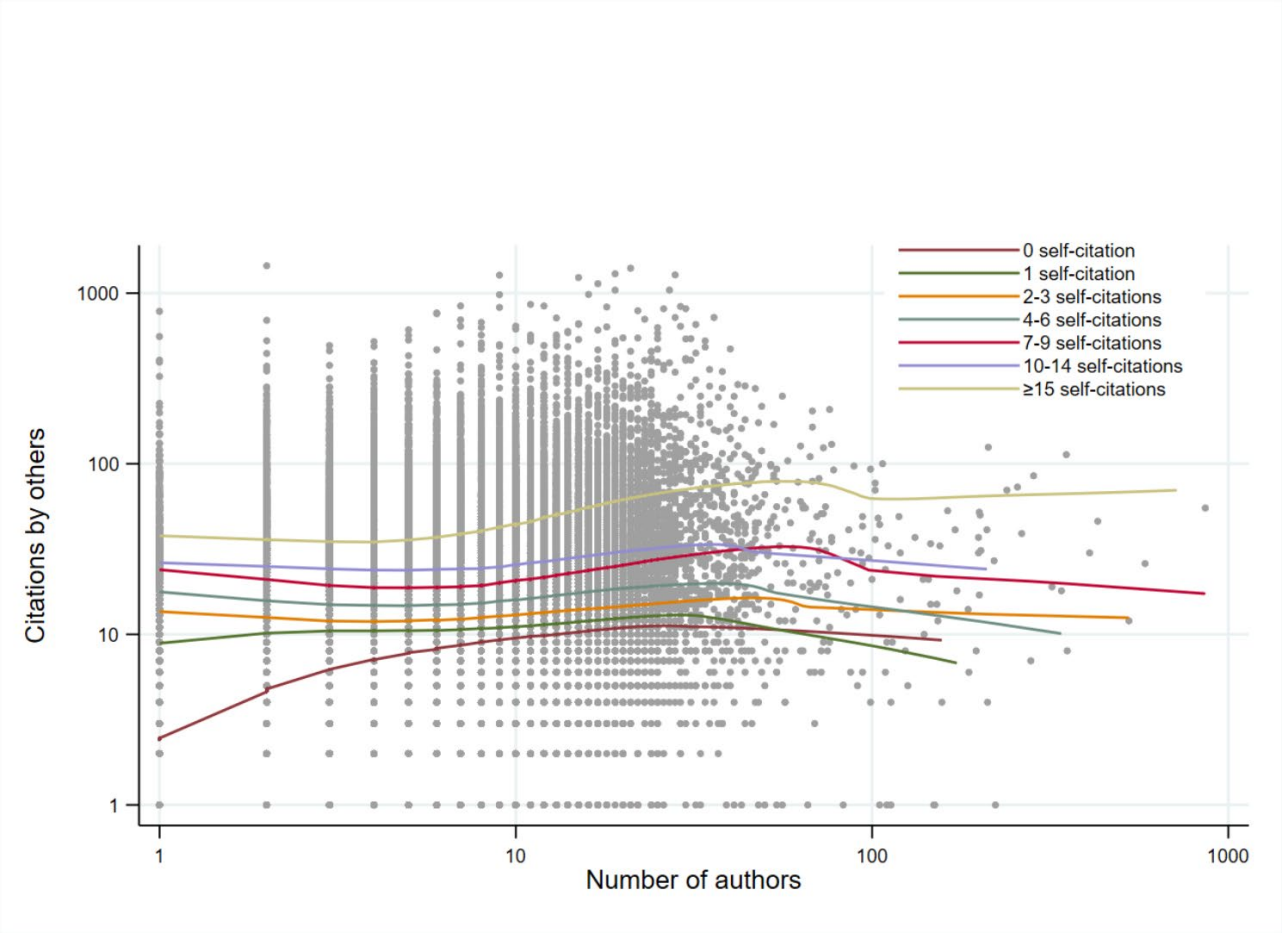
Scatter-plot of the number of citations by others (logarithm scale) as a function of the number of authors (logarithm scale), stratified by the number of collective self-citations. Lines are non-parametric regression functions (Lowess)

We also explored the associations between the number of authors and each type of citation graphically, using non-parametric functions to represent the associations. Collective self-citations increased gradually, almost linearly, as the number of authors increased, within each stratum of citations by others (Fig. 2). In contrast, the number of citations by others increased much less as the number of authors increased, within each stratum of collective self-citations (Fig. 3).

### Collective self-citations and citation thresholds

Because the h-index, a commonly used metric in research assessment, is based on the number of articles that exceed a certain number of citations, we examined the impact of collective self-citations on the proportion of articles that will reach or exceed specified thresholds, namely 10, 20, 30 or 50 citations (Table 2). The impact of collective self-citations strongly depended on the number of authors. For example, the threshold of 30 citations (of any type) was reached or exceeded by 8.4% of single-author articles, 17.4% of articles written by 2–4 authors, and up to 60.9% of articles written by 30–49 authors. If only citations by others were counted, these proportions ranged from 7.7 to 49.3%. Thus, collective self-citations increased the proportion of articles receiving ≥ 30 citations by 0.7% for single authored articles, 3.7% for articles written by 2–4 authors, and up to 11.6% for articles written by 30–49 authors. Of note, collective self-citations alone would suffice to attain 30 citations for 0.1% of single author articles, but 21.1% of articles written by ≥ 50 authors.

**Table 2:** Proportions (in %) of articles reaching or exceeding thresholds of citations, for total citations, citations by others, collective self-citations, and increase in the proportion due to collective self-citations, for 88,594 articles in health-related fields published in 2015

	①	②	③	① - ②
Number of authors	All citations	Citations by others	Collective self-citations	Increase from collective self- citations
	**Proportion with at least 10 citations**			
1	28.4	25.9	1.5	2.5
2–4	59.8	51.5	4.9	8.3
5–9	70.1	59.8	8.7	10.3
10–19	80.0	69.6	17.5	10.3
20–29	90.7	82.8	37.8	8.0
30–49	90.6	83.3	49.7	7.3
≥ 50	85.8	74.7	52.6	11.1
	**Proportion with at least 20 citations**			
1	15.2	13.3	0.3	1.9
2–4	31.0	25.3	0.8	5.8
5–9	37.8	29.3	1.4	8.5
10–19	51.3	40.4	4.2	10.9
20–29	70.6	58.8	16.3	11.8
30–49	75.4	62.9	28.9	12.4
≥ 50	67.9	56.3	30.0	11.6
	**Proportion with at least 30 citations**			
1	8.4	7.7	0.1	0.7
2–4	17.4	13.8	0.2	3.7
5–9	21.0	15.6	0.3	5.4
10–19	32.6	24.6	1.5	7.9
20–29	54.2	44.7	8.0	9.4
30–49	60.9	49.3	14.9	11.6
≥ 50	56.8	45.3	21.1	11.6
	**Proportion with at least 50 citations**			
1	3.8	3.4	0.0	0.4
2–4	6.7	5.4	0.0	1.3
5–9	8.0	5.9	0.1	2.0
10–19	15.5	11.6	0.3	3.9
20–29	34.6	27.5	2.6	7.1
30–49	43.2	32.2	7.5	11.0
≥ 50	41.6	25.3	7.4	16.3

## Discussion

In this analysis of citation outcomes of scientific articles in the health sciences, we observed that the number of authors of a scientific article was more strongly associated with collective self-citations than with citations by others, as suggested by the contrast in the association patterns between self-citations and citations by others after mutual adjustment. If we accept that citations by others are an indicator of overall scientific utility, then some other mechanism must explain why the association between the number of authors and collective self-citations is stronger. Our hypothesis is that this simply reflects the size of the pool of potential self-citers, who have their own motivations (independent of scientific utility) for citing their own work. Importantly, this analysis does not address the issue of whether collective self-citations are justified or not. Citing one’s works has a range of reasons [5], from strictly technical (such as citing a previously developed method), to the wish to assert one’s credibility in the field vis-à-vis editors, reviewers and readers; gaming research assessment indicators is a possibility but not a necessary presumption. Because some of those reasons pertain to scientific utility, excluding all self-citations from calculation could lead to underestimating a paper’s utility. We believe that an h-index value truly reflecting one author’s production of scientifically utile knowledge lies somewhere between one calculated using all collective self-citations (overestimate) and one excluding all collective self-citations (underestimate). We also examined the potential impact of collective self-citations on threshold-based metrics such as the h-index. Collective self-citations increased the proportions of articles that reached or exceeded a given citation threshold more strongly for multi-authored articles than for articles written by few authors. The discrepancy was greater for higher citation targets than for lower citation targets (e.g., ≥ 30 citations vs. ≥ 10 citations). This suggests that collective self-citations by multiple authors may have a substantial impact on the h-index of each of them, and this was reported in other studies [23, 24]. A previous study of academic radiologists which suggested that the impact of collective self-citations or individual self-citations on the h-index was minimal [25], but this study analyzed individual self-citations and not collective self-citations.

Limitations of the present study include the difficulties in accurately identifying the same author across different publications. Although Scopus assigns a unique identifier to each author, we cannot exclude that one could have been identified as a new author after re-registering with another affiliation or omitting one’s middle name. We would expect, however, that this happens randomly across papers of varied number of co-authors, thus not altering our results fundamentally. Secondly, not all collective self-citations are driven by motives other than scientific utility. As a result, and as mentioned above, subtracting all collective self-citations from a total citation score might underestimate the scientific utility of a paper as some, maybe a great portion of them, might be scientifically motivated. Thirdly, we chose a 5-year follow-up to allow enough citations to accrue. A longer follow-up might have strengthened the observed patterns; however, this would have pushed back the publication year, and the data used might have been seen as not relevant to current practice. This decision means that our results are only generalizable to the citations made in the five years following a publication and we cannot exclude that the effect of the number of authors changes after that, although we believe it to be unlikely. Finally, increasing the number of authors on a publication de facto reduces the number of external individuals who could potentially cite it. Although we do not believe that this is of significance for common research topic, this could play a role for very niche research fields where a larger team would leave fewer ‘others’ to potentially cite the study.

Can collective self-citations associated with long author lists distort research assessment? We believe they can, because collective self-citations do not necessarily reflect the utility of the work for the broader scientific community. This would not be a problem if collective self-citations helped all papers to the same extent, but the benefit is clearly greater for multi-authored papers, independently from how useful they seem to be by the broader scientific community. Thus, authors who repeatedly participate in multi-authored studies will attain higher h-index values than authors who collaborate with fewer co-authors, even if citations by others were the same. While scientific collaborations are valuable, it is questionable whether a collaborative publication should bring more credit to a researcher than a publication with few co-authors, if citations by others are the same.

What are the implications of these results? We would recommend that h-index be reported along with its alternative calculation which disregards collective self-citations. This would be helpful for the assessment of both individual researchers (e.g., by promotions committees or funding agencies), research institutions, and for journal-level citation indicators. Counting only citations by others would protect such indicators from any distortion caused by multiple authorship, and would also discourage willful manipulation by some authors. While manipulation may be rare, its mere possibility casts a shadow on citation analyses globally. Currently, citation databases such as the Web of Science or Google Scholar do not provide analyses based solely on citations by others, but implementing such results should not be too difficult, especially if unique personal identifiers such as the Orcid number become commonplace. This would be a step towards a more objective assessment of researchers’ contributions to their scientific field.

### Electronic supplementary material

Below is the link to the electronic supplementary material.

Supplementary Material 1

## Data Availability

With the authorization of the International Center for the Study of Research (ICSR) Lab which powers Scopus’ solutions, the data are being shared along with this publication. They are accessible at the following address: 10.26037/yareta:n5n5c2ndqvdnzfhqosdaefef2y.
