# Correction: In health research publications, the number of authors is strongly associated with collective self-citations but less so with citations by others

**DOI:** 10.1186/s12874-023-02084-3

**Published:** 2023-11-07

**Authors:** Cyril Jaksic, Angèle Gayet-Ageron, Thomas Perneger

**Affiliations:** 1 UAM-CRC & Division of Clinical Epidemiology, Department of Health and Community Medicine, University of Geneva & University Hospitals of Geneva, Geneva, Switzerland; 2 Present address: Hôpitaux Universitaires de Genève, Service d’épidémiologie Clinique, Bvd de La Tour 8, 1211 Geneva, Switzerland; 3 Division of Clinical Epidemiology, Department of Health and Community Medicine, University of Geneva & University Hospitals of Geneva, Geneva, Switzerland

**Correction: BMC Med Res Methodol 23, 230 (2023)**

**https://doi.org/10.1186/s12874-023-02037-w**

Following publication of the original article [1], the authors identified an error in Table 1. The first column "Number of authors" has been updated. The correct table is given below.

**Table 1:** Distribution of research articles across 7 categories of numbers of authors, and means (SD) and medians (Q1-Q3) of citations obtained in 2015–20 (Total citations, citations by others, collective self-citations, self-citations as percentage of total), and percentage of uncited papers, for 88,594 articles in health-related fields published in 2015

Number of authors	N (%)	Total citations, mean (SD) median (Q1-Q3)	Citations by others, mean (SD) median (Q1-Q3)	Collective self-citations, mean (SD) median (Q1-Q3)	Percent collective self-citations, mean (SD) median (Q1-Q3)	Uncited articles (percent)
1	2618 (3.0)	10.5 (29.8) 2 (0—11)	9.6 (28.8) 2 (0—11)	0.8 (2.5) 0 (0—11)	10.6 (20.1) 0 (0—11)	40.6
2–4	19306 (21.8)	19.9 (76.5) 12 (6—23)	17.3 (75.9) 10 (4—23)	2.6 (3.8) 1 (0—23)	16.5 (19.7) 10 (0—23)	6.2
5–9	43159 (48.7)	22.5 (81.6) 15 (8—26)	18.9 (80.6) 12 (6—26)	3.6 (4.8) 2 (1—26)	19.3 (19.4) 14 (4—26)	1.4
10–19	20956 (23.7)	32.4 (54.6) 20 (11—36)	26.7 (50.2) 15 (8—36)	5.7 (7.7) 4 (1—36)	21.4 (18.5) 17 (8—36)	0.5
20–29	1874 (2.1)	67.2 (157.9) 33 (18—69)	55.5 (145.4) 26 (13—69)	11.6 (16.7) 7 (3—69)	23.2 (17.6) 20 (11—69)	0.1
30–49	491 (0.6)	109.4 (347.0) 39 (20—87)	91.0 (325.8) 29 (14—87)	18.4 (31.3) 9 (4—87)	26.0 (18.0) 23 (12—87)	0.6
≥ 50	190 (0.2)	93.7 (339.4) 36 (15—71)	72.5 (305.4) 26 (9—71)	21.2 (39.4) 10 (4—71)	34.8 (22.8) 32 (19—71)	0.5

The original article [1] has been updated.
