# Supplementary material for: In health research publications, the number of authors is strongly associated with collective self-citations but less so with citations by others
Source: BMC Med Res Methodol. 2023 Oct 11;23:230. doi: 10.1186/s12874-023-02037-w (PMC10568899; doi:10.1186/s12874-023-02037-w)
Supplement: Supplementary file 1 — Supplementary Material 1 [file 12874_2023_2037_MOESM1_ESM.docx]

Appendix A

List of the ASJC codes, and their associated field, retained for the present study.

| **ASJC** | **Field** |
| --- | --- |
| 2700 | General Medicine |
| 2701 | Medicine (miscellaneous) |
| 2703 | Anesthesiology and Pain Medicine |
| 2705 | Cardiology and Cardiovascular Medicine |
| 2706 | Critical Care and Intensive Care Medicine |
| 2707 | Complementary and Alternative Medicine |
| 2708 | Dermatology |
| 2711 | Emergency Medicine |
| 2712 | Endocrinology, Diabetes and Metabolism |
| 2713 | Epidemiology |
| 2714 | Family Practice |
| 2715 | Gastroenterology |
| 2717 | Geriatrics and Gerontology |
| 2720 | Hematology |
| 2721 | Hepatology |
| 2723 | Immunology and Allergy |
| 2724 | Internal Medicine |
| 2725 | Infectious Diseases |
| 2726 | Microbiology (medical) |
| 2727 | Nephrology |
| 2728 | Neurology (clinical) |
| 2729 | Obstetrics and Gynecology |
| 2730 | Oncology |
| 2731 | Ophthalmology |
| 2732 | Orthopedics and Sports Medicine |
| 2733 | Otorhinolaryngology |
| 2735 | Pediatrics, Perinatology and Child Health |
| 2736 | Pharmacology (medical) |
| 2738 | Psychiatry and Mental Health |
| 2739 | Public Health, Environmental and Occupational Health |
| 2740 | Pulmonary and Respiratory Medicine |
| 2741 | Radiology, Nuclear Medicine and Imaging |
| 2742 | Rehabilitation |
| 2743 | Reproductive Medicine |
| 2745 | Rheumatology |
| 2746 | Surgery |
| 2747 | Transplantation |
| 2748 | Urology |
| 2909 | Gerontology |
| 2916 | Nutrition and Dietetics |
| 2919 | Pediatrics |
| 2921 | Psychiatric Mental Health |
| 3504 | Oral Surgery |
| 3604 | Emergency Medical Services |
| 3609 | Occupational Therapy |
| 3611 | Pharmacy |
| 3612 | Physical Therapy, Sports Therapy and Rehabilitation |
| 3615 | Respiratory Care |
